# The Etiologic Relationship of Focal Infections to Systemic Disease

**Published:** 1915-01

**Authors:** Noble Wiley Jones

**Affiliations:** Portland, Oregon


### THE ETIOLOGIC RELATIONSHIP OF FOCAL INFECTIONS TO SYSTEMIC DISEASE.

BY NOBLE WILEY JONES, M.D., PORTLAND, OREGON.

A year ago, through the courtesy of your program committee, I read a paper before your society on the common and erroneous ideas of the relationship of oral sepsis and the swallowing of pus to gastro-intestinal diseases, anemias, malnutrition, etc.; but owing to the lack of time I merely mentioned in a subsidiary way some of the common diseases which had been proved to arise from different focal pus infections. Your second invitation gives me the opportunity to epitomize briefly these latter proved etiologic facts, the establishment of which form one of the most epochal and far reaching advances made in medicine in the last twenty years.

It is largely through the early work of Poynton and Payne in acute articular rheumatism and their persistent claim that a specific micro-organism (micrococcus rheumaticus) was the cause of this disease, and the early immunological studies in chronic pneumococcus endocarditis by Schottmueller, Horder, Libman, Rosenow and others, that the present understanding of focal infections and their relation to certain common systemic diseases of uncertain origin has had its birth. Any circumscribed and confined infection constitutes a focal infection, wherever it may be located in the body. The usual location is about or in the tonsil, the gums and alveolar processes, the sinuses and the middle ear; but a chronically infected gall bladder, appendix, fallopian tube, seminal vesical, prostate, toe nail, or localized abscess anywhere, has been shown, in specific instances, to be primarily the starting place of general infections. Furthermore, a secondary focus of infection in a regional lymph gland may ultimately act as the primary focus in maintaining the general infection.

By a study of the infected tissues with the newer methods of tissue culture of Rosenow, various pathogenic bacteria have been isolated. Staphyococci, the varying strains of streptococci and pneumococci, and gonococci are the most important ones. Bacillus coli and Bacillus aerogenous capsulatus occur, seemingly as mixed infections, in such varied locations as joint tissues and regional lymph glands, the gall bladder, the glands of Hodgkin's disease, exopthalmic goitre and ovarian cysts; but their real significance is not yet understood.

It is quite probable that the only way in which an infection from a given focus becomes systemic, is through the lymph and blood streams. In animal experiments it is only by direct inoculation of the blood stream that the diseases in question have been reproduced. The tissues concerned in the morbid process, namely, the heart valves, the submucosa of the stomach and duodenum, the gall bladder, the tendon sheaths, joint capsules, etc., have functionaly, at least, end arteries, and the histology of the lesions is that of an embolic infraction with local hemorrhage and endothelial proliferation, which closes the lumen of the vessel. The invading bacteria becomes sealed up in an area of necrosis, largely out of reach of the various protective substances in the body sera. Arthritis deformans and chronic infectious endocarditis are good examples of this method of self-protection on the part of the infectious agent, although it exists in the two conditions in widely differing degrees of oxygen tension. These mico-organisms anatomically immunize themselves against the body sera and resist all efforts at treatment.

Last year Dr. Rosenow (one of the co-workers of Dr. Billings in Chicago) published his studies on the transmutations within the streptococcus-pneumococcus group, a work which is worthy a Pasteur or a Koch and which will remain epochal in this decade of modern medicine. His work, in brief, has proven that the various strains of streptococci and pneumococci are modifications of one parent type of micro-organism brought about as a response to altered environment. This alteration of environment has largely to do with a change in oxygen tension, so that by cultural procedures and animal passage a streptococcus with certain characteristics and affinities may be transformed into other types with other characteristics and affinities and again into pneumococci, or vice versa. In the main there are five types of micro-organisms belonging to this group which may be transmuted back and forth: First, the hemolytic streptococcus which is lowest in virulence and has greatest affinity for the joints and tendon sheaths; second, the pneumococcus with a high degree of virulence and affinity for especially the lungs; third, streptococcus viridans, a more or less saprophitic, non-hemolysing streptococcus with especial affinity for the endocardium; fourth, the streptococci from acute rheumatism which partake of the affinities of the hemolytic and viridans streptococci and explain the frequency of heart lesions in the course of an acute rheumatism, and, fifth, the streptococcus mucosus which has some of the properties of both pneumococci and streptococci. The micrococcus rheumaticus of Poynton and Payne, the diplococcus rheumaticus of Beatty and the streptococcus rheumaticus of others have thus been harmonized and explained by these studies. Furthermore, the significance of the focus of infection has been broadened so that it no longer can be looked upon as merely the place of entrance of the organisms, but *"as the place where they acquire the affinities necessary to infect."* From the observations of especially Billings and Davis the focus is most commonly found in the oral cavity, and the order in frequency is probably first the tonsil, secondly pyorrhoea and thirdly alveolar abscess.

The great array of disease entities which have been proved or are now suspected of having an etiologic relationship to focal infections reads like a page of an address by Sir W. Arbouthnot Lane on chronic intestinal statis. But in most of the following diseases this relationship has been scientifically proven and are not the bedside deductions of a brilliant surgical mind. There is no doubt of the etiologic factor of focal infections in acute rheumatism, arthritis deformans, gonorrhoeal arthritis, myositis, infectious endocarditis and various septicemias and visceral degenrations. There is strong evidence that especially ulcer of the stomach and duodenum is due almost wholly to oral infections. In twenty-five thyroid glands out of thirty-two cases of goitre removed at operation (most of them being exopthalmic in type) an anaerobic Gram-positive diplo-bacillus like organism was isolated by Rosenow; and the same was found in eight out of twelve dogs with goitre. This organism injected intravenously into two healthy dogs resulted in goitre; so that the assumption is warranted that goitre may be due to the localization of bacteria in the gland as well as to toxins. Pancreatitis, cholecystitis and acute nephritis have been shown to be related to focal infections in certain instances. Some years ago Eppinger noted clinically the good effect of removing infected tonsils in acute nephritis following tonsilitis.

Of particular interest to me has been the result of Rosenow's experimental production of stomach and duodenal ulcers by the intravenous injection of streptococci of low virulence. In his transmutation studies Rosenow noted that ulcer was frequently found in animals after injection of a streptococcus with a virulence a little higher than that possessed by the hemolytic streptococcus which produces rheumatism. When the virulence increased and approached that of the pneumococcus or streptococcus viridans, ulcer did not occur. From the bases of excised ulcers from the human stomach a similar streptococcus with a similar low degree of virulence was repeatedly isolated by him and this organism injected into animals produced again the ulcers in the stomach and duodenum very frequently. Since the first of June of this year I have studied clinically about twenty cases of ulcer of the stomach and duodenum and every one of them has had a marked history of tonsillar or dental sepsis. I have seen one case of recurrence of ulcer after excision of the primary ulcer in a patient in whom an intense pyorrhea had been neglected after operation.

In concluding this brief résume of recent work on focal infections in general and of dental sepsis in particular, I wish again to emphasize as I did before you last year the fact that both dentists and physicians have not yet awakened sufficiently to the importance of this subject. Too many patients pass through the consulting room of the physician without having his mouth examined at all. Far too many patients pass through your offices without a word of advice or caution about the existence of a pyorrhoea, and dentists are still placing crowns and bridges upon infected stumps and creating often times out of a pussing gum with free drainage a sealed pus cavity.

Last week I referred a man for dental work into whose mouth a year and a half ago a dentist had placed an entire upper and partial lower set of gold crowns and bridges over the broken and decayed remains of teeth that fairly reeked with pus. Such a dentist may easily condemn a patient to a living death through subsequent arthritis or heart disease. I am convinced that the average dentist regards the loss of a tooth almost as a doctor might the loss of a patient, and in this spirit the dentist usually has the willing co-operation of his patient as well. Fortunately for everybody concerned the life insurance companies are now recognizing the importance of these examinations. Already applicants are being refused insurance on the ground of oral infections. This fact will in itself cause many doctors to stop and seriously consider the question. I hope it may also cause the dentists to take better notice, too. There is not enough co-operation between these two professions. It is not logical nor fitting that they should exist as such either, for dentistry should be a specialty of the practice of medicine, the same as opthalmology or orthopoedic surgery. This is the case in Germany where the graduated dentist must have first a preliminary education in medicine together with its degree. It would be presumptious on my part to enumerate to you the signs of dental sepsis, for you should recognize them quicker than I. But because some of you do not I hope you will permit me to suggest that you have in mind always the general condition of your patient when occasion demands it at all and inquire about his general health, the presence of rheumatic pains, of stiff and painful joints, shortness of breath on slight exertion, distress after eating, etc., and if any doubt exists as to the possibility of sealed pus pockets or non-erupted teeth which might be infected, to make free use of the X-ray and make sure.

### DISCUSSION BY ARTHUR W. CHANCE, D.D.S.

The essayist has said that this further discovery of focal infections being the cause of hitherto unknown causes of many diseases, marks an epoch in medical discoveries. I believe that it marks the *dawn* of what dentistry may be and what, if we will fully grasp its significance, dentistry is sure to be; that is, the prime element in the cure of some of the worst diseases that afflict mankind. We stand on the threshold of a new era in dental practice. We are just about to realize the ambitions of dentists for the past seventy-five years and, if we equip ourselves to take advantage of this new knowledge, we shall soon stand shoulder to shoulder with medicine, law, the ministry, and all learned professions. For as soon as the knowledge of the dangers of septic mouths and the advantages of hygienic oral cavities becomes common, full value for the services to mankind will be given the dentist who can carry out conscientiously the principles of aseptic surgical procedure which suggest themselves in hearing this valuable paper.

We must recognize and cure pyorrhea in our patients. Or, of more importance to their welfare, we must prevent its occurrence. And, as we all know, that the foundation of much of our reparative work upon the teeth depends upon absolute success in root canal work, we must develop a scientific mode of procedure in devitalizing pulps, treating infected root canals and alveolar abscess and filling the canals of devitalized teeth as to be sure of our results, if we are to meet the responsibilities which these new discoveries in medicine put upon us. We must, therefore, give up those empirical practices of using "pusturine," "pus-cure" and "oxypara," which are so widely advertised and so frequently used without exact knowledge of their formula or their effects upon human tissue. If we do not inform ourselves more fully upon pathology, bacteriology, and improve our technic, we shall be forced by a knowing medical profession and later a fully informed public to extract teeth that are dangerous to general health and thus destroy our usefulness in helping to provide an efficient masticating apparatus for our patients by preserving their natural teeth.

The American dentist has been known the world over for his mechanical skill. Such discoveries in medicine as the essayist has brought to our attention should make us students of more than the mechanical repair of lost tooth structure with which most of our work has been heretofore, and we should see to it that we get a broader knowledge of disease, its causes and its cures than mere mechanical treatment requires.

But with it all do not let us become convinced, as many recent dental writers would have us believe, that *all* constitutional diseases have their origin in the mouth and in the mouth alone. For it is well to remember that other seats for possible infective foci, such as the tonsils, the sinuses, the fallopian tubes, and indeed, abscesses anywhere, still exist in the human body.

That we may not fully disappoint a great surgeon. who, in a recent address, mentioned the possibilities of preventive medicine in our field of dentistry, let us then become better informed upon the modern fundamental facts of health and disease and so adapt our technic as to preserve the one and assist the brilliant minds in medicine and surgery in the cure of the other.—*Northwestern Dental Journal.*

